# Phase II study of UFT and oxaliplatin in first-line treatment of advanced colorectal cancer

**DOI:** 10.1038/sj.bjc.6602217

**Published:** 2004-10-26

**Authors:** J Feliu, J M Vicent, C García-Girón, M Constela, E Fonseca, J Aparicio, M Lomas, L Antón-Aparicio, F J Dorta, M Gonzalez-Baron, and Oncopaz Cooperative Group and Associated Hospitals

**Affiliations:** 1 Medical Oncology Service, Hospital La Paz de Madrid, P° de la Castellana, Madrid 261-28046, Spain; 2 Medical Oncology Service, Hospital General Universitario, Valencia, Spain; 3 Medical Oncology Service, Hospital General Yagüe, Burgos, Spain; 4 Medical Oncology Service, Complejo Hospitalario, Pontevedra, Spain; 5 Medical Oncology Service, Hospital Universitario, Salamanca, Spain; 6 Medical Oncology Service, Hospital La Fé, Valencia, Spain; 7 Medical Oncology Service, Hospital Infanta Cristina, Badajoz, Spain; 8 Medical Oncology Service, Hospital Juan Canalejo, La Coruña, Spain; 9 Medical Oncology Service, Hospital Nta. Sra. De la Candelaria, Santa Cruz de Tenerife, Spain

**Keywords:** colorectal cancer, chemotherapy, UFT, oxaliplatin, leucovorin

## Abstract

The purpose of this study was to evaluate the efficacy, assesed as response rate, and toxicity of UFT (Tegafur-Uracil) in combination with oxaliplatin as first-line treatment of advanced colorectal cancer (CRC). In all, 84 patients with recurrent or metastatic CRC with measurable disease were included. Treatment consisted of oxaliplatin 85 mg m^−2^ in 120-min intravenous (i.v.) infusion on days 1 and 15; i.v. l,leucovorin (l,LV) 250 mg m^−2^ given in 2 h on day 1, followed by oral UFT 390 mg m^−2^ on days 1–14, and oral l,LV 7.5 mg/12 h on days 2–14. Cycles were repeated every 28 days. A total of 492 cycles of chemotherapy were delivered with a median of six per patient (range 1–12). There was one complete response (1%) and 28 partial responses (34%) for an overall response rate of 35% (95% confidence interval (CI): 24–46%). A total of 36 patients (44%) had stable disease, whereas 17 (21%) had a progression. The median time to progression was 7.3 months and the median overall survival was 16.8 months. A prescheduled preliminary analysis was performed after inclusion of 16 patients who detected a high gastrointestinal toxicity, which led to a reduction of the UFT dose to 300 mg m^−2^. With this new dosage, grade 3–4 diarrhoea and grade 3–4 nausea/vomiting dropped to 21 and 14% of patients, respectively. Other grade 3–4 toxicities were stomatitis in one (1%), anaemia in three (5%), neutropenia in two (3%), thrombocytopenia in one(1%), fatigue in six (9%), peripheral sensory neuropathy in nine (14%) and laryngopharyngeal dysesthesia in two patients (2%). The combination of oxaliplatin and UFT–l,LV is an active, easy-to-administer regimen with moderate toxicity. Hence, this regimen is worthy of further investigation.

Although 5-fluorouracil (5FU) was developed approximately 50 years ago, it is still an essential part of advanced colorectal cancer (CRC) treatment. It is currently accepted that 5FU continuous infusion and biochemical modulation with leucovorin (LV) increase response rates and time to progression compared to schemas with bolus administration (Advanced Colorectal Meta-Analysis Project, 1992; Meta-Analysis Group in Cancer, 1998a). They also have a more favourable toxicity profile (Meta-Analysis Group in Cancer, 1998b).

Over the last 10 years, significant activity has been observed with other cytotoxics, such as irinotecan (CPT-11) and oxaliplatin, in monotherapy treatment of this tumour type. The addition of oxaliplatin or CPT-11 to 5FU–LV in randomised Phase III trials has shown high antitumoral activity in patients with advanced CRC (de Gramont *et al*, 2000; Douillard *et al*, 2000; Giacchetti *et al*, 2000; Saltz *et al*, 2000).

Although combined therapy has been an important development in the treatment of advanced CRC, these schemes do have some disadvantages. Firstly, their increased toxicity, particularly when CPT-11 is combined with bolus 5FU–LV (Ledermann *et al*, 2001; Rothenberg *et al*, 2001). Secondly, continuous-infusion-based regimens require the use of implantable access devices and portable infusion pumps. In addition to the potential risk of catheter-related complications, this type of regimen can restrict patient activity, which can have a negative effect on their quality of life. For this reason, oral chemotherapy may represent a convenient and more acceptable treatment modality. Besides, some studies indicate that patients prefer oral rather than intravenous (i.v.) therapy, provided that efficacy remains the same (Liu *et al*, 1997; Borner *et al*, 2002).

UFT is an oral combination of uracil and tegafur in a molar ratio of 4 : 1. Tegafur is a prodrug that is metabolised into 5FU by hepatic microsomal cytochrome P450 enzymes or by ubiquitous cytosolic enzymes. Uracil inhibits the catabolism of 5FU by competitive inhibition of the enzyme dihydropyrimidine-dehydrogenase, which maintains active drug levels for a prolonged period and thus simulates a continuous infusion of 5FU. The plasma half-life is of 5–12 h (Fujii *et al*, 1979; Eng *et al*, 2001). In a pharmacokinetic study, UFT 370 mg m^−2^ given orally on a 28-day schedule resulted in blood concentrations comparable to those following a continuous i.v. infusion of 5FU 250 mg m^−2^ (Ho *et al*, 1998). Two Phase III studies have been conducted to compare the efficacy and toxicity of modulation of UFT and LV with 5FU–LV (Carmichael *et al*, 2002; Douillard *et al*, 2002). Response rate and overall survival were similar, although a slight disadvantage in time to progression was observed for UFT–LV in one of the studies (3.8 *vs* 3.5 months; *P*<0.05) (Douillard *et al*, 2002). However, if we take these studies as a whole, it is acceptable to say that they are equivalent in efficacy and that UFT–LV has a more favourable toxicity profile, with less neutropenia, diarrhoea, nausea/vomiting and mucositis (Mayer, 2001).

Some years ago, we developed a therapeutic scheme to modulate UFT with LV. It consisted of the infusion of a high dose of LV, followed by the oral administration of both UFT and LV for 14 days.

A Phase I trial determined that the maximum-tolerated dose of UFT when modulated in this way was 390 mg m^−2^ day^−1^ (González-Barón *et al*, 1993). This regimen obtained a 39% response rate in patients with advanced colon cancer, both adults (González-Barón *et al*, 1995) and elderly patients (Feliu *et al*, 1997).

Oxaliplatin is a third-generation platinum compound that has synergistic antitumoral activity with 5FU (Raymond *et al*, 1997). This synergistic activity is also observed when oxaliplatin is combined with UFT–LV (Louvet *et al*, 2000).

Therefore, in the light of the above, it is to be expected that the combination of oxaliplatin–UFT–LV will be at least as effective as the combination of oxaliplatin–5FU–LV, although the former could offer greater comfort for patients and a lower toxicity.

The purpose of this multicentre Phase II study is to assess the efficacy (in terms of response rate) and safety of the oxaliplatin–UFT–LV combination as first-line treatment in patients with advanced CRC.

## PATIENTS AND METHODS

### Patient population

A total of 84 patients with recurrent or metastatic CRC were included from April 1999 to January 2000. They all had at least one lesion histologically or cytologically confirmed adenocarcinoma. Patients who had received prior adjuvant 5FU-based chemotherapy were eligible if they had remained free of disease for at least 6 months after completion of the adjuvant therapy. Patients with operable metastatic disease were excluded from the study. Other inclusion criteria were: (1) a performance status ⩽2, according to Eastern Cooperative Oncology Group (ECOG) scale. (2) Life expectancy of at least 3 months. (3) Adequate bone marrow function, that is, a granulocyte count ⩾2 × 10^9^ l^−1^ and platelets >100 × 10^9^ l^−1^. (4) Adequate hepatic function, that is, serum bilirubin <1.25 times the upper normal limit, glutamic oxaloacetic transaminase values (SGOT) and glutamic pyruvic transaminases (SGPT) < 2.5 times the upper normal limit in the absence of hepatic metastases or <5 times the upper normal limit in the presence of metastasis. (5) Adequate renal function, that is, a creatinine value ⩽1.25 times the upper normal limit.

Patients with any prior chemotherapy for advanced disease, brain or meningeal metastases, or a history of any other malignancy were excluded, except in cases of basal cell carcinoma or *in situ* cervical carcinoma adequately treated. Patients provided written informed consent according to directives of local ethical committees.

All patients had measurable disease, as defined by the presence of at least one bidimensionally measurable lesion by computed tomography scan. Pleural effusion, ascites, osteoblastic lesions or previously irradiated lesions were not accepted as measurable disease. Patients who had received radiotherapy were eligible if there was at least one measurable lesion outside the radiation field.

### Treatment plan

The study regimen consisted of oxaliplatin 85 mg m^−2^ in 120-min i.v. infusion in 250 ml of dextrose 5% on days 1 and 15; i.v. l,LV 250 mg m^−2^ given over 2 h on day 1, followed by oral UFT 390 mg m^−2^ on days 1–14; and oral l,LV 7.5 mg 12 h^−1^ on days 2–14, followed by 2 weeks of rest. An entire course lasted 4 weeks. Pills were taken before meals to favour absorption (for instance, at 0800 and 2000). For practical reasons, UFT doses were rounded up or down to the nearest dose that could be administered with 100 mg capsules of the drug. Routine antiemetic prophylaxis with a 5-hydroxytryptamine-3-receptor antagonist was used. Courses were repeated every 28 days with a minimum of three per patient, unless progressive disease was detected. Responding patients continued therapy until progression or the appearance of unacceptable toxicity.

Patients were assessed for toxicity before each course and graded according to WHO scales (WHO, 1979). Complete blood counts were obtained on days 1 and 14 of each cycle, before each administration of oxaliplatin. Therapy was delayed for 1 week if the neutrophil count was <1.5 × 10^9^ l^−1^ or the platelet count was <100 × 10^9^ l^−1^ or for significant persisting nonhaematologic toxicity. Therapy was completely discontinued if toxicity persisted after a 2-week delay. In case of grade 3 or 4 haematologic toxicity or any other severe (⩾grade 3) organ toxicity, the dose of all drugs was decreased by 25% in the subsequent cycles. The oxaliplatin dose was reduced by 25% for subsequent cycles in case of persistent (⩾14 days) or temporary (7–14 days) painful paresthesia or functional impairment. In case of persistent painful paresthesia or functional impairment, or if a patient experienced any other severe neurotoxicity despite a 25% dose attenuation, oxaliplatin was omitted in subsequent cycles. In case of the occurrence of a laryngeal spasm syndrome, the duration of the oxaliplatin infusion was increased from 2 to 6 h. In case of persistent problems, oxaliplatin was omitted.

### Pretreatment and follow-up studies

Patients had a full clinical history, physical examination, performance status assessment, haematological and biochemical profiles (including CEA level), a chest X-ray and a computed tomography scan of the chest and abdomen at baseline. Additional imaging investigations were performed if clinically indicated. A computed tomography scan was repeated every three courses to assess objective response. At the end of chemotherapy, all clinical, laboratory and imaging studies were repeated and patients underwent follow-up examination every 2 or 3 months until death.

### Toxicity and response criteria

Toxicity for each course was recorded and graded according to WHO scales (WHO, 1979). For toxicity analysis, the worst data for each patient across all courses were used. Response was evaluated using WHO guidelines (WHO, 1979). A complete response required the total disappearance of all tumours initially observed in two observations not less than 4 weeks apart, with no evidence of new areas of malignant disease. A partial response was defined as a reduction of at least 50% in the sum of the products of the longest perpendicular diameter of all clearly measurable tumour masses, in two observations not less than 4 weeks apart, with no increase in the size of any lesion and no evidence of new lesions. Stable disease was defined as a decrease in total tumour size of less than 50% or a less than 25% increase in any measurable lesion. Progression was defined as a 25% increase in the size of any lesion, the appearance of new areas of malignant disease or performance status deterioration by more than one level. Time to tumour progression was estimated by the product-limit estimation from the date of the first course to the first evidence of disease progression. Radiological responses were not evaluated by an Independent Review Committee (IRC). Survival was calculated by the same method from the date of the first course until the date of death or last known follow-up.

### Statistical analysis

The primary end point was response rate and the secondary objectives were survival and time to progression. Dose intensity was calculated by dividing the total mg m^−2^ of drug given by the number of weeks elapsed from the beginning of therapy to the end of the last cycle.

The sample size was designed to reject a response rate of less than 20%. A total of 19 patients were initially included using the Fleming method (Fleming, 1982). Alternatively, a planned sample size of 80 evaluable patients was chosen to better estimate efficacy; 20% of the maximum width of the 95% confidence interval (CI) for an expected 35% overall response rate. The Wilcoxon's rank-sum method was used to compare quantitative variables, the Fisher's exact test for percentages and the Kaplan–Meier method for survival and the duration of response. Progression-free survival was measured from the start of chemotherapy to the date of progressive disease or death without progression.

## RESULTS

### Patient characteristics

A total of 84 patients with recurrent or metastatic CRC were included on the study. The characteristics of these patients are shown in Table 1

**Table 1:** Patient characteristics

**Characteristic**	**No. (%)**
Age (years) (mean and range)	63 (40–77)

*Sex*	
Male	47 (56%)
Female	37 (44%)
	
*ECOG performance status*	
0	41 (49%)
1	37 (44%)
2	6 (7%)
	
*Primary site*	
Colon	52 (62%)
Rectum	32 (38%)
	
*Metastasis sites*	
Liver	62 (74%)
Lung	25 (30%)
Local abdominal mass	16 (19%)
Others	19 (23%)
	
*No. of metastatic sites*	
1	47 (56%)
2	32 (38%)
⩾3	5 (6%)
	
*Adjuvant chemotherapy*	
Yes	30 (36%)
No	54 (64%)

. The median age of the series was 63 years (range 40–77). In total, 47 patients were male (56%) and 37 were female (44%). There were 41 patients (49%) with an ECOG performance status of 0, 37 (44%) with 1 and 6 (7%) with 2. In 52 patients (62%), the primary tumour was located in the colon and in 32 (38%) in the rectum. A total of 22 patients (26%) had previously received chemotherapy as adjuvant therapy and other eight (10%) had received chemotherapy and radiotherapy. The liver was the predominant metastatic site (74%), and the median number of involved sites was two per patient.

### Treatment summary

A total of 492 cycles of chemotherapy were delivered with a median of six cycles per patient (range 1–12). Eight patients (10%) received less than three cycles of chemotherapy: four (5%) due to a progression, two (2%) due to the patient's refusal and a further two (2%) because they moved to another city and were lost to follow-up. With the exception of the last two patients, for whom no data are available, the remaining 82 patients were evaluated for efficacy and toxicity. The first 16 patients received UFT 390 mg m^−2^ day^−1^ but, because of the high toxicity associated with this dose, the subsequent 66 patients received 300 mg m^−2^ day^−1^.

The median dose intensity of UFT was 1020 mg m^−2^ week^−1^ in the first group (83% of the scheduled dose) and 934 mg m^−2^ week^−1^ in the second group (89% of the scheduled dose). The median dose intensity of oxaliplatin was 38.2 mg m^−2^ week^−1^, which corresponded to 90%. A total of 69 patients (82%) received 90% or more of the scheduled dose.

### Response and survival

Response data are listed in Table 2

**Table 2:** Therapeutic results in 82 patients

**Results**	**No. of patients (%)**
Complete response	1 (1%)
Partial response	28 (34%)
Stable disease	36 (44%)
Progressive disease	17 (21%)

Overall response 35% (95% CI: 24–46%).

. One patient (1%) obtained a complete response and 28 (34%) had a partial response, with an overall response rate of 35% (95% CI: 24–46%). A total of 36 patients (44%) had stable disease and 17 (21%) had a progression. The median follow-up for all patients was 14 months. The median duration of response was 7.8 months. The median time to progression was 7.3 months, and the median overall survival 16.8 months. The 1-year actuarial survival rate was 71%.

### Toxicity

The first 16 patients received UFT 390 mg m^−2^ day^−1^. Important gastrointestinal toxicity was observed with grade 3–4 diarrhoea in nine patients (56%) and grade 3–4 nausea/vomiting in three (19%). One patient developed grade 3 neutropenia (6%) (Table 3

**Table 3:** Toxicity by patients

	**UFT 390 mg m^−2^, 16 pts.**		**UFT 300 mg m^−2^, 66 pts.**	
	**Grade 1–2**	**Grade 3–4**	**Grade 1–2**	**Grade 3–4**
Nausea/vomiting	12 (75%)	3 (19%)	32 (48%)	9 (14%)
Diarrhoea	4 (25%)	9 (56%)	28 (42%)	14 (21%)
Stomatitis	4 (25%)	0	9 (14%)	1 (1%)
Gastric pain^a^	3 (19%)	0	5 (8%)	0
Neutropenia	3 (19%)	1 (6%)	18 (27%)	2 (3%)
Thrombocytopenia	10 (62%)	0	32 (48%)	1 (1%)
Anaemia	9 (56%)	0	32 (48%)	3 (5%)
Asthenia	12 (75%)	4 (25%)	25 (38%)	6 (9%)
Sensitive neuropathy	10 (62%)	3 (18%)	39 (59%)	9 (14%)
Transaminases	6 (37%)	0	22 (33%)	0

a Gastric pain: Grade 1–2: abated with antacids or H2-blockers. Grade 3–4: intense enough to require withdrawal of UFT.

). As a result, the UFT dose was reduced to 300 mg m^−2^ in subsequent patients. This reduced dose was administered to 66 patients and the incidence of grade 3–4 diarrhoea and nausea/vomiting dropped to 21 and 14% of patients, respectively. This toxicity appeared preferentially in the first two cycles. Other grade 3–4 toxicities were stomatitis in one patient (1%), anaemia in three (5%), neutropenia in two (3%), thrombocytopenia in one (1%) and fatigue in six (19%). Oxaliplatin-associated peripheral sensory neuropathy was observed in 73% of patients, with grade 3 neurotoxicity occurring in nine (14%) patients. Grade 3–4 laryngopharyngeal dysesthesia was observed in two patients (2%), and could be prevented in the following treatment cycles by prolonging the duration of oxaliplatin infusion.

## DISCUSSION

Advanced CRC is currently an incurable disease. Therefore, the objective of treatment must be to prolong survival, obtain effective control of symptoms and maintain or improve quality of life. These objectives should be achieved with an acceptable level of toxicity that does not deteriorate the patient's quality of life.

At present, many treatments used in advanced CRC are based on continuous infusion of 5FU modulated by LV, combined with oxaliplatin or CPT-11. However, continuous infusions require an indwelling central venous catheter, which is a potential risk for infection and thrombosis. Moreover, continuous infusions increase treatment costs and probably reduce patient quality of life. Therefore, the development of schemes with oral fluoropyrimidines that can replace 5FU continuous infusion effectively is an area of research to pursue given the potential enhanced convenience and cost benefits. The aims of our study were to obtain the same therapeutic results as 5FU–LV–oxaliplatin, but with greater convenience for the patient by avoiding the use of infusion pumps. The results obtained show that the combination UFT–l,LV–oxaliplatin is relatively effective in advanced CRC, with overall response rates of 35% and a median survival rate of 16.8 months. Although the response rate is somewhat lower than the 45–60% response rate reported with other oxaliplatin–5FU–LV schemes in first-line therapy of advanced CCR (Bertheault-Cvitkovik *et al*, 1996; de Gramont *et al*, 2000; Giacchetti *et al*, 2000; Goldberg *et al*, 2003), it does not differ significantly from those reported in different Phase II studies with oxaliplatin–capecitabine (31–55%) (Shields *et al*, 2002; Makatsoris *et al*, 2003; Tabernero *et al*, 2003; Zeuli *et al*, 2003). On the other hand, time to progression and median survival rate are similar to those obtained with these combinations (Bertheault-Cvitkovik *et al*, 1996; de Gramont *et al*, 2000; Giacchetti *et al*, 2000; Shields *et al*, 2002; Louvet and de Gramont, 2003; Makatsoris *et al*, 2003; Simpson *et al*, 2003; Tabernero *et al*, 2003; Zeuli *et al*, 2003). Notwithstanding, it can be mentioned that radiological responses were not reviewed by an IRC, and this could have overestimated our results. In all events, in the absence of Phase III trials with which we can compare these schemes directly, we should use these comparisons with caution.

With regard to toxicity, we should point out the high levels of gastrointestinal toxicity obtained with the initially planned dose of UFT 390 mg m^−2^, as 56% of the patients who received this dose developed some form of grade 3–4 toxicity. After reducing the UFT dose to 300 mg m^−2^ in the subsequent patients, the incidence of grade 3–4 diarrhoea fell to 21% of patients and grade 3–4 nausea and vomiting to 14%. These figures are similar to those reported with 5FU–LV–oxaliplatin schemes (Simpson *et al*, 2003), although the latter reported a higher frequency of grade 3–4 neutropenia (41–48% of patients) (de Gramont *et al*, 2000; Giacchetti *et al*, 2000) than that detected in our study (3%).

Relatively few authors have investigated the combination of oxaliplatin–UFT with or without LV, in comparison to the association of oxaliplatin–capecitabine. In a Phase II study on 34 patients previously treated with a fluoropyrimidine-based regimen, oxaliplatin 130 mg m^−2^ was administered every 21 days with UFT doses of 350 mg m^−2^ for 21 days, without modulation by LV. The response rate was 13% and grade 3–4 toxicity was exceptional, with just one episode of Grade 3 neutropenia (Kim *et al*, 2002). In another Phase II trial that included 64 patients, oxaliplatin 130 mg m^−2^ was administered with UFT (300 mg m^−2^) for 2 weeks, modulated by LV. An overall response rate of 34% and a median time to progression of 5.9 months were obtained, with 12% of patients developing grade 3–4 neutropenia, 11% grade 3 diarrhoea and 8% suffering from grade 3 nausea/vomiting (Douillard *et al*, 2004). These data coincide with what we have observed in our series.

We could conclude by saying that the results of our study suggest that UFT/l,LV and oxaliplatin can be combined safely. The recommended dose for future studies is UFT 300 mg m^−2^, days 1–14, l,LV 250 mg m^−2^ i.v. on day 1, l,LV 15 mg day^−1^, days 2–14 and oxaliplatin 85 mg m^−2^ on days 1 and 14, with repeat treatments every 4 weeks. This is an active scheme with a 35% response rate and a median survival of 16.5 months. It has acceptable toxicity and offers the advantage of convenience with its oral administration. Hence, this regimen is worthy of further investigation.
